# Asymmetric Organocatalysis: A Survival Guide to Medicinal Chemists †

**DOI:** 10.3390/molecules28010271

**Published:** 2022-12-29

**Authors:** Efraim Reyes, Liher Prieto, Andrea Milelli

**Affiliations:** 1 Department of Organic and Inorganic Chemistry, University of the Basque Country (UPV/EHU), 48080 Bilbao, Spain; 2 Department for Life Quality Studies, Alma Mater Studiorum-University of Bologna, Corso d’Augusto 237, 47921 Rimini, Italy

**Keywords:** asymmetric organocatalysis, chirality, chiral drugs, drug discovery, drug synthesis

## Abstract

Majority of drugs act by interacting with chiral counterparts, e.g., proteins, and we are, unfortunately, well-aware of how chirality can negatively impact the outcome of a therapeutic regime. The number of chiral, non-racemic drugs on the market is increasing, and it is becoming ever more important to prepare these compounds in a safe, economic, and environmentally sustainable fashion. Asymmetric organocatalysis has a long history, but it began its renaissance era only during the first years of the millennium. Since then, this field has reached an extraordinary level, as confirmed by the awarding of the 2021 Chemistry Nobel Prize. In the present review, we wish to highlight the application of organocatalysis in the synthesis of enantio-enriched molecules that may be of interest to the pharmaceutical industry and the medicinal chemistry community. We aim to discuss the different activation modes observed for organocatalysts, examining, for each of them, the generally accepted mechanisms and the most important and developed reactions, that may be useful to medicinal chemists. For each of these types of organocatalytic activations, select examples from academic and industrial applications will be disclosed during the synthesis of drugs and natural products.

## 1. Introduction

Most of the prescribed drugs exert their activity by interacting with a biochemical counterpart that, given its spatially defined three-dimensional shape, is chiral. Therefore, this recognition process is highly stereospecific. This simple word has had a massive impact in all the phases of today’s drug discovery process, from the hit-discovery phase to the fine-tuning of the manufacturing process [1]. Despite the infamous thalidomide case related to the chirality and stereospecificity of the drug–biological counterpart interaction, up until 20 years ago pharmacopoeias were still dominated by racemic drugs, and only in 1992 did the Regulatory Agencies start to set some guidelines for commercializing chiral drugs [2]. Crucial in this context was the Food and Drug Administration document entitled “Development of new stereoisomeric drugs”, which states that, amongst other things, “unless it proves particularly difficult, the main pharmacologic activities of the isomers should be compared in in vitro systems, in animals and/or in humans” [2]. This has represented a point of no return for the drug discovery industry. Indeed, based on the report from Agranat et al. [3] in the decade 2001–2010, out of 195 new molecular entities approved by the FDA, 108 were single enantiomers. Importantly, such a significant switch was possible thanks to the development of suitable technological platforms allowing the synthesis and/or the separation of a given chiral compound in substantial quantities, as well as the identification of the chiral compounds. Concerning the preparation of enantiopure compounds, three main approaches can be followed [4]: (a) resolution of racemates, (b) synthesis from the chiral pool, and (c) synthesis from prochiral substrates. In the latter case, the chiral information is mainly transferred from an enantiopure catalyst to a non-chiral compound. Among the different catalysts available for such transformation, in the last twenty years we have observed a stunning and unanticipated development of a peculiar type of catalysis known as ***asymmetric organocatalysis***. This outbreak was certified by the chemical community and came to the public’s attention as best as it could.

Indeed, on 6 October 2021, a press release from the Swedish National Academy of Sciences announced David W. C. MacMillan and Benjamin List as the recipients of the 2021 Noble Prize in Chemistry for the development of asymmetric organocatalysis. Central to the prize assignment was the declaration “using these reactions, researchers can now more efficiently construct anything from new pharmaceuticals to molecules that can capture light in solar cells. In this way, organocatalysts are bringing the greatest benefit to humankind”.

**Figure 1:**
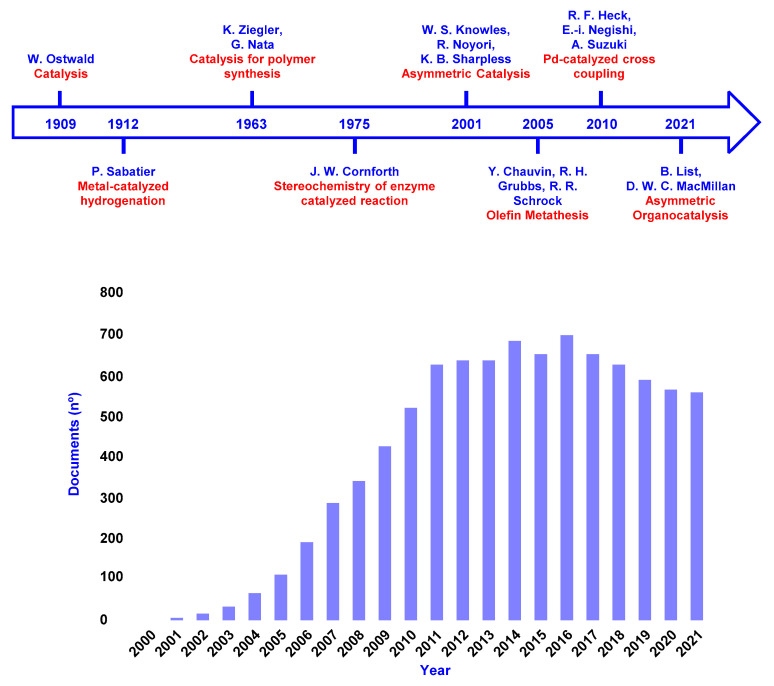
Historical timeline of the Nobel Prize in Chemistry devoted to catalysis (**upper part**) and the number of articles containing “organocatalysis” or “organocatalytic” in the title, abstract, or keywords per year (Scopus search, October 2022). Reviews, book chapters, editorials, and conference papers are not included (**bottom part**).

This announcement had a great impact on the chemistry community since asymmetric organocatalysis was a relatively young research field and the previous Noble Prize recognizing catalysis in organic chemistry was awarded only ten years earlier (Figure 1).

Asymmetric organocatalysis is, by definition [5], the use of small organic molecules to accelerate reactions, inducing stereochemical information. It represented a disruptive conceptual shift in the mind of chemists that were used to dealing with metal compounds or biomolecules as catalytic systems. The success of organocatalysis derives from the several advantages it has compared to enzymes and metal catalysts. Indeed, although enzymes are safe to use and work splendidly in a physiological environment, they are very expensive, do not work well under normal organic conditions of solvent, temperature, and so on, and they can be highly specific with respect to substrate and, thus, can suffer from a very limited scope. On the other hand, metal catalysts, although highly efficient, are often hazardous compounds to both humans and the environment and should, therefore, be carefully eliminated from the final commercialized material. In addition, their disposal poses significant issues. They are often not compatible with air or moisture and, therefore, their use requires special conditions that can be very expensive and demanding to achieve in industrial plants. Based on the advantages inherent with stable and easy to handle organocatalysts, there are no surprises that, nowadays, almost all universities have research groups devoting their area of interest to asymmetric organocatalysis, and plenty of industrial syntheses, especially concerning drug manufacturing, have many key steps using organocatalysis. 

The beginning of this new area of research started when both Macmillan [6] and List [7] published their seminal papers in 2000 and, since then, there has been an explosive growth in the field, considering the importance of chiral molecules in our daily life. A Scopus search (October 2022) for articles containing “organocatalysis” or “organocatalytic” in the title, abstract, or keywords retrieved the impressive number of more than 9000 entries since the year 2000 (Figure 1).

**Figure 2:**
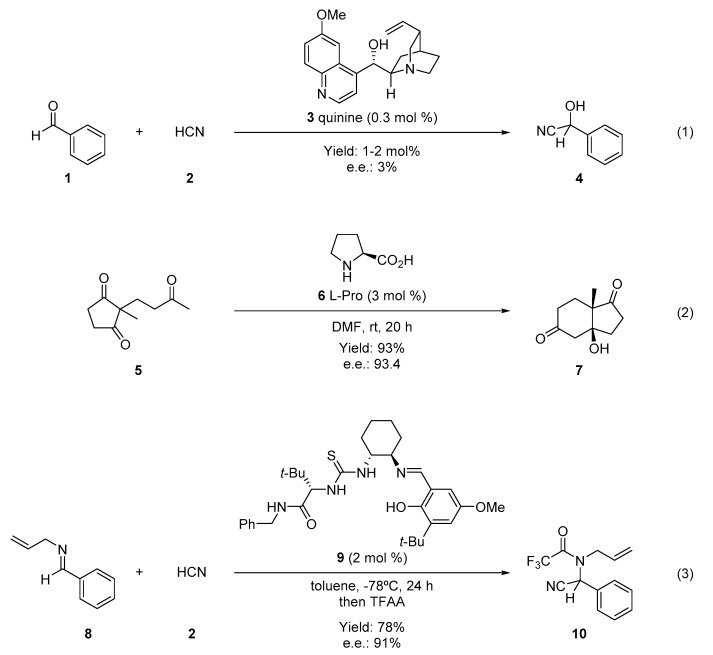
Examples of preliminary asymmetric organocatalytic reports (1900–1999).

However, the reports by MacMillan and List were not the first examples of organocatalytic reactions that appeared in the literature; indeed, several reactions that fulfill the given definition for organocatalysis were reported years before, even if they appeared as isolated examples without a general conceptualization. For example, the first report about the use of a small organic molecule to asymmetrically catalyze an organic transformation was back in 1912, when Bredig and Fiske showed that chiral cinchona alkaloids (**3**) may catalyze the addition of hydrogen cyanide (**2**) to benzaldehydes (**1**) to obtain cyanohydrines (**4**), although in low enantiomeric excess (Figure 2(1)) [8]. Another pivotal study was reported in 1971 when Hajos and Parrish recognized that L-proline (**6**) catalyzed the intramolecular cyclization of ketotrione **5** to furnish the Wieland–Miescher ketone **7** in high yield and enantiomeric excess (e.e.) (Figure 2(2)) [9,10]. More recently, in 1998 Jacobsen and coworkers first reported that a thiourea-based catalyst (**9**) acts using hydrogen-bonding to catalyze an asymmetric Strecker reaction, leading to the adduct **10** in high yield and high e.e. (Figure 2(3)) [11]. 

Interestingly, in the 1990s, the groups of Barbas III and Lerner generated catalytic antibodies, mimics of a class I aldolase, that use the primary amine of a lysine residue to catalyze an aldol reaction [12,13,14]. A retrospective analysis by Prof. Barbas even suggested that organocatalysis might have been the “key chemistry that could enable the asymmetric prebiotic synthesis of the building block of life” [15]. Notwithstanding these reports, why the field of organocatalysis had not been conceptualized before the year 2000 is not easy to elucidate. Indeed, MacMillan, in his commentary published in 2008, pointed out that even “Dieter Seebach, an organic chemist of great standing, omitted organocatalysis from his vision for synthetic methods in 1990” [5]. The simultaneous and independent publications by List and Barbas and by MacMillan represented the breakthrough that finally conceptualized the field and triggered the explosion of asymmetric organocatalysis. Since that moment, organocatalysis has become the third pillar of asymmetric catalysis, providing chemists with a spectacularly powerful tool for the synthesis of enantio-enriched compounds.

In the present review, we wish to highlight the application of organocatalysis in the synthesis of enantio-enriched molecules that may be of interest to the pharmaceutical industry and the medicinal chemistry community [16,17]. In our review, we aim to discuss the different activation modes for the organocatalytic asymmetric carbon–carbon bond forming reactions examining the general mechanisms, the most important reactions, and the use in medicinal chemistry by selecting examples from academic and industrial research directed toward the synthesis of drugs and/or natural products. 

A large number of research articles and reviews have been published in the organocatalysis field during the last several years [18,19,20,21,22,23,24,25,26,27,28,29,30,31]. However, most of them are highly specialized and, in most of the cases, their reading assumes a profound knowledge of the subject. Our intention is not to provide a comprehensive overview of organocatalysis but, rather, to provide a general “tutorial” of the field and a critical overview about the possibilities that organocatalysis can open up in the design and synthesis of new chemical entities. This perspective is aimed at those who are approaching this topic for the first time, such as graduate students or chemists from industry or academy who have never had to deal with this topic.

## 2. Generic Activation Modes

**Figure 3:**
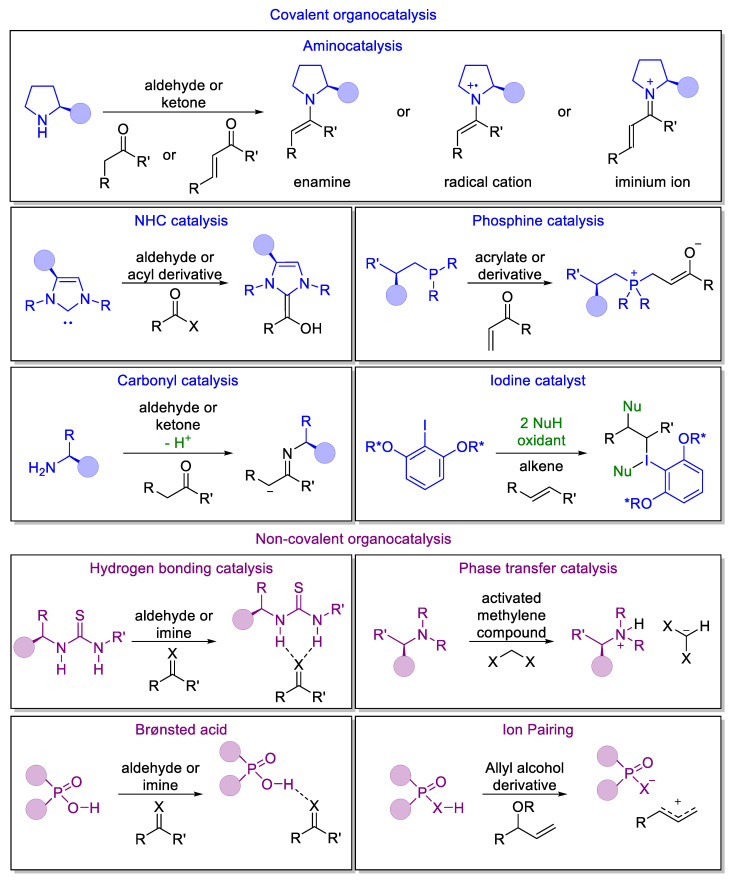
Main organocatalytic activation modes: In covalent activation, the most used activation mechanism, relying on aminocatalysis (enamine, single-occupied molecular orbital (SOMO), and iminium ion catalysis), NHC, phosphine catalysis, carbonyl catalysis, and iodine catalysis. In noncovalent activation, the main activation modes are based on hydrogen-bond, phase-transfer, Brønsted acid, and ion-pairing catalysis.

There are several reasons behind the success of organocatalysis, including the wide availability, low cost, and safety of organocatalysts, as well as the simplicity of the procedure required to perform an organocatalyzed reaction. However, perhaps the most important characteristic of this type of catalysis is the identification of generic modes of catalyst activation and reactivity, leading to a wide range of possible transformations achievable with these catalysts. Organocatalysts activate different substrates mainly following two different patterns: covalent and noncovalent activation (Figure 3). 

Covalent-based activation [32] modes are based on the formation of a reactive intermediate thanks to the creation of a covalent reversible interaction between a catalyst and a substrate. Covalent activation mainly relies on aminocatalysis. Other important activation modes directed to forming a C-C bond rely on the use of nitrogen heterocyclic carbenes (NHC) [33,34,35,36,37,38,39,40], phosphines [41,42], carbonyl compounds [43,44], and iodine derivatives [45] as catalysts. Although those catalysts show interesting properties, aminocatalysis represents the principal activation mode in organocatalysis due to the possibility of recycling of the catalyst, scaling up the reaction, etc. Importantly, chiral secondary amines can activate a carbonyl compound, i.e., saturated or unsaturated aldehydes or ketones, via the formation of a nucleophilic enamine (for enamine, dienamine, or trienamine catalysis), an electrophilic iminium ion (for iminium catalysis), or a radical cation (for SOMO catalysis) (see later). It is important to point out that, and this is one of the reasons behind the success of organocatalysis, a single organocatalyst can promote all these different activation modes, leading to a multifunctional catalyst.

In noncovalent catalysis [46], the activation of a substrate occurs via weak interactions, such as hydrogen-bonding (hydrogen-bond catalysis or Brønsted acid catalysis) or ionic interactions (phase-transfer catalysis and ion-pairing). Hydrogen-bonding catalysis [47,48] and Brønsted acid catalysis [49,50,51,52,53,54] activate electrophiles toward a nucleophilic attack. This powerful synthetic strategy is abundantly used in nature since a discrete number of enzymes, such as serine proteases, exert their catalytic functions using hydrogen-bond activation. Phase-transfer catalysis [55,56,57,58], pioneered by the asymmetric alkylation methodology developed at Merck in 1984, allows carbon–carbon and heteroatom–carbon bond formation under mild biphasic conditions. Through H-bonding catalysis, several substrates may be activated, and this is not restricted only to carbonyl compounds, and therefore, different reaction types have been established, including rearrangements and cyclizations. Thanks to the versatility of these activation modes, over the years several different classes of catalyst have been developed, including thioureas and diols. In the case of ion-pairing catalysis [59,60,61], the catalyst acts by donating a hydrogen to an electronegative acceptor, leading to the formation of a counterion pair. It is important to note that phosphoric acid and related catalysts can act through this last mechanism or through a simple H-bonding catalysis. 

## 3. Enamine Catalysis

**Figure 4:**
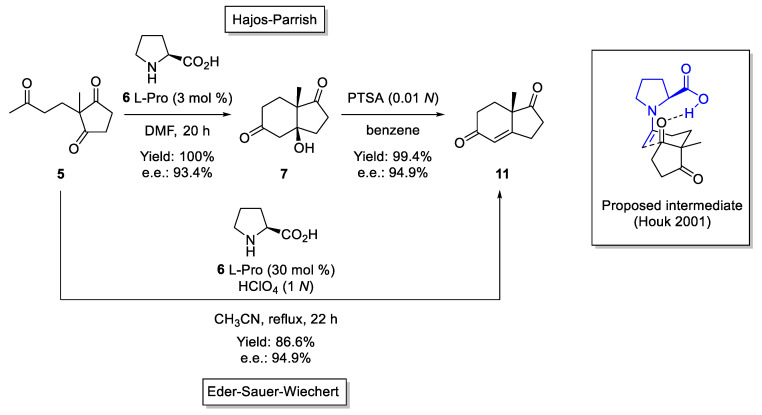
The Hajos–Parrish–Eder–Sauer–Wiechert reaction.

The utility of enamines as carbon nucleophiles has been well-known since the middle of the last century when Prof. G. Stork and coworkers at Columbia University described the alkylation and acylation of different enamines that were easily generated from ketones and secondary amines [62]. In these reactions, enamines acted as enol surrogates, providing an indirect method for the functionalization of ketones. This methodology was also studied in other reactions in which the enamines act as C-nucleophiles, including Michael and aldol reactions. In this sense, the stoichiometric use of secondary amines for converting ketones into enamines and for using the latter during the indirect functionalization at the α-carbon was the method of choice for many organic transformations. Years later, a special secondary amine, L-Proline (**6**), was used to generate enamines, both stoichiometrically and catalytically, during the synthesis of Miescher ketone **11** through an intramolecular aldol reaction, the so-called Hajos–Parrish–Eder–Sauer–Wiechert reaction (Figure 4) [9]. Computational study of this reaction proposed a chiral enamine as the reactive intermediate [63,64], allowing the utilization of this methodology in several enantioselective synthesis of steroids [65].

**Figure 5:**
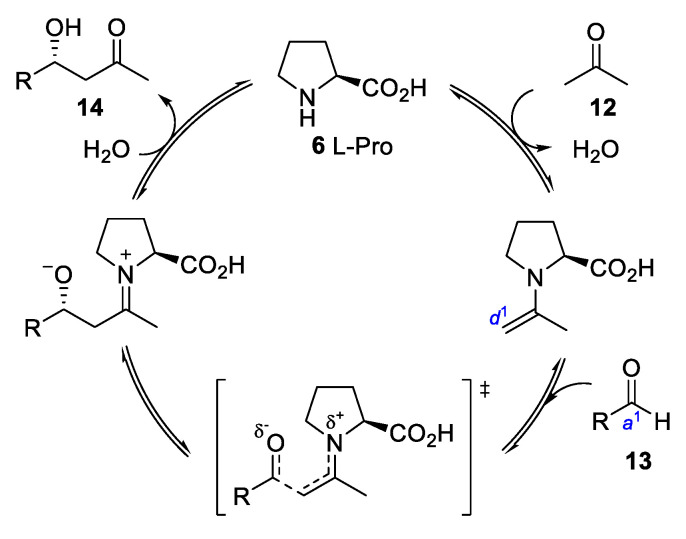
Simplified catalytic cycle for the intermolecular aldol reaction using acetone **4**.

The corresponding catalytic enantioselective, intermolecular aldol reaction, that is, the nucleophilic addition of a ketone (acetone, **12**) to differently substituted aldehydes (**13**), was presented in 2000, marking the initial point for asymmetric organocatalysis [7]. Similar to the Stork reaction in which an enamine is used stoichiometrically, the accepted catalytic cycle starts with the condensation of the ketone **12** with the catalyst, the secondary amine L-Proline (**6**), to form the corresponding enamine (Figure 5). This acts as a *d*^1^ nucleophile reacting with an aldehyde (**13**) (*a*^1^ electrophile), thus generating the desired aldol product (**14**) after hydrolysis, which liberates the amino acid that enters in a new catalytic cycle.

**Figure 6:**
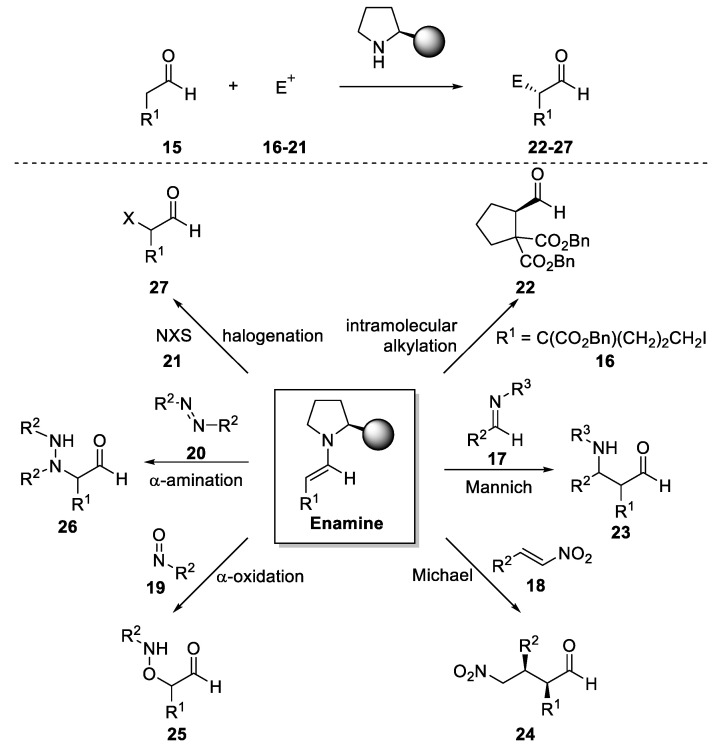
Some of the possible α-functionalization of an enamine intermediate.

The operational simplicity of this reaction together with the high yields and enantioselectivities observed has led to the extension of this methodology to other reactions in which an enamine acts as the nucleophile [66]. In this sense, a number of catalysts have been used for performing α-functionalization of carbonyl compounds (**15**) (aldehydes and ketones), such as intramolecular alkylations, Mannich reactions, Michael additions, α-oxidations, α-sulphenylations, α-aminations, α-halogenations, etc., using the different electrophilic species (**16**–**21**) (Figure 6).

**Figure 7:**
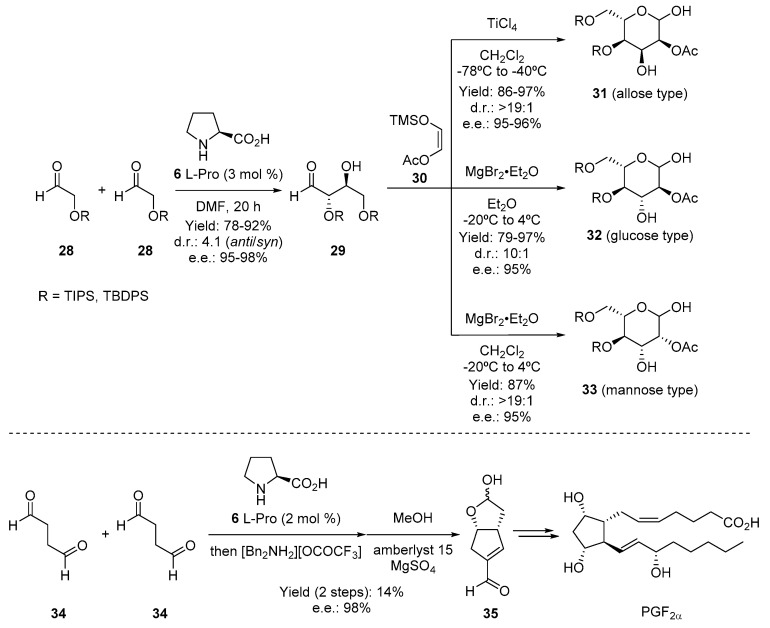
(**Top**) Two_step carbohydrates’ synthesis developed by MacMillan and coworkers. (**Bottom**) Synthesis of prostaglandin PGF_2α_ by means of organocatalytic aldol reaction.

Among the potential applications uncovered by using organocatalysis as the main tool, the cross-aldol reaction between different aldehydes (**28**) represents one of the most relevant processes in the area. This reaction is important since it gives direct access to aldol products, very useful compounds for the preparation of polyketides, such as **29**. In this sense, Macmillan and coworkers presented in 2002 the two-step synthesis of sugar derivatives **31**–**33** by using the catalytic enantioselective cross-aldol reaction as the key step [67,68]. With this simple methodology, several sugar-type skeletons can be synthesized in a direct manner (Figure 7 top). The cross-aldol reaction, again using L-Proline (**6**) as a catalyst, has also been studied during the preparation of prostaglandin PGF_2α_ in seven steps (Figure 7 bottom) [69]. The key step relies on the preparation of the bicyclic compound **35**, which was obtained in a relatively low yield but with excellent stereoselection. This compound could also be obtained in large quantities for continuing the synthesis through simple chemical modifications.

**Figure 8:**
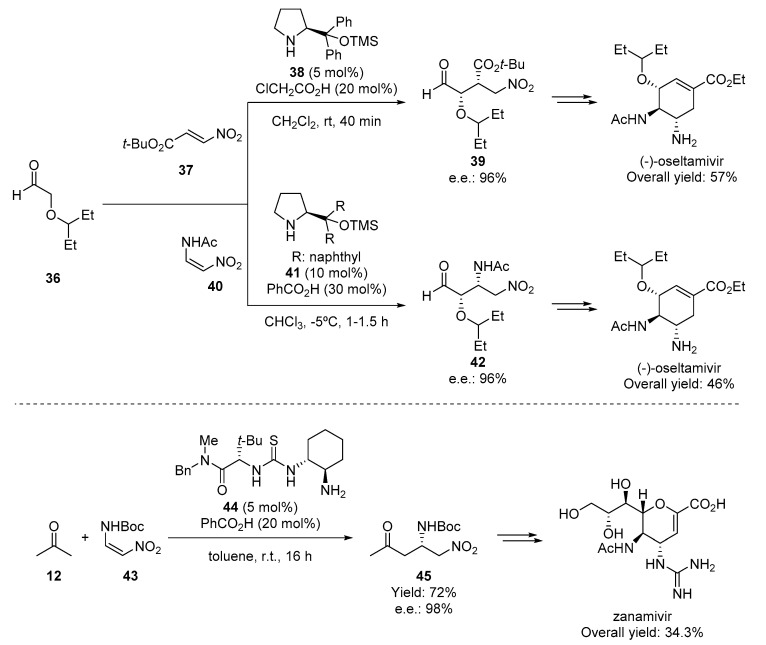
Organocatalytic Michael reactions allowing the synthesis of oseltamivir and zanamivir.

As depicted in Figure 6, one of the most important features of the secondary amine-mediated catalysis is the possibility of performing other types of reactions under similar reaction conditions. Thus, exposing the enamine to a Michael acceptor give access to α-functionalized aldehydes or ketones through a simple Michael-type reaction. This methodology has been used for the preparation of the influenza antiviral agents, oseltamivir [70,71] and zanamivir [72], by using the nitroalkenes **37**, **40**, and **43** as Michael acceptors, respectively (Figure 8). Whereas in the first case, the Jørgensen–Hayashi [73] catalysts **38** and **41** (an L-proline derivative) were used for the preparation of the key Michael adducts **39** and **42**, with almost complete enantioselectivity, the synthesis of zanamivir used the bifunctional primary amine catalyst **44** for performing a similar reaction using acetone **12** as the starting material.

These and other examples illustrate the importance of enamine catalysis during the preparation of complex structures with high levels of enantioselectivities. Furthermore, the usefulness of this type of catalysis is mainly due to compatibility with other types of catalysis, including iminium [74,75,76], NHC [77], and metal catalysis [78], by providing a useful platform for the rapid functionalization of simple starting materials, exploiting the principle of vinylogy by performing remote functionalization [79]. 

## 4. Iminium-Ion Catalysis

**Figure 9:**
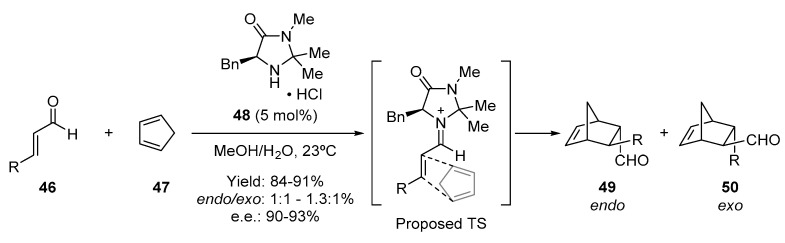
The first organocatalytic Diels–Alder reaction, introduced by MacMillan.

Soon after the publications of List, Lerner, and Barbas III re-discovering enamine catalysis, MacMillan and coworkers reported the discovery of iminium-ion catalysis that used the secondary amine **48** as a Diels–Alder-mediated catalyst for performing the aforementioned cycloaddition, in this case between an α,β-unsaturated aldehyde (**46**) and a diene (**47**) [7]. This discovery introduced the “iminium-ion” concept as a general activation mode in asymmetric synthesis (Figure 9). In this reaction, an unsaturated iminium-ion is the catalytically generated dienophile. The structure of the catalyst controls the approach of the diene from the less hindered face, which results in almost complete control of the enantioselectivity in the formed adduct.

**Figure 10:**
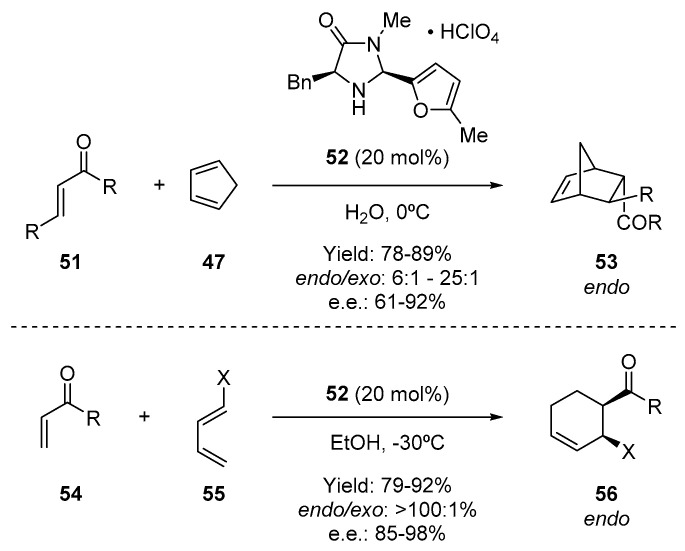
Organocatalytic Diels–Alder reaction employing ketones as dienophiles.

Iminium-ion catalysis turned out to also be a powerful methodology to activate α,β-unsaturated ketones as dienophiles in a similar Diels–Alder reaction (Figure 10) [80]. In this case, a new imidazolidinone (**52**) was employed as a catalyst and the reaction proceeded with high stereoselectivity with both acyclic and cyclic ketone **51** and **54**, with diverse dienes **47** and **55**, respectively. With these examples, MacMillan demonstrated the utility of these new types of catalysts in highly important cycloaddition reactions.

**Figure 11:**
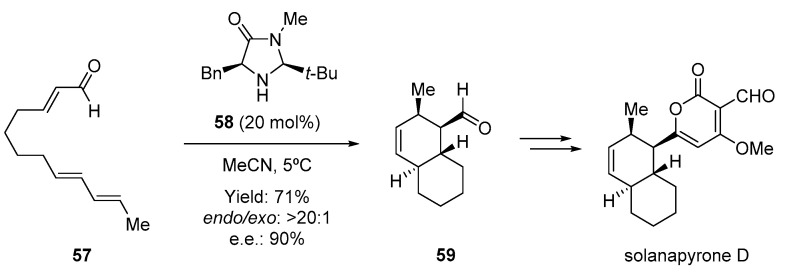
Application of the intramolecular Diels–Alder reaction in the synthesis of Solanapyrone D.

As mentioned earlier, this methodology initiated a new area of research in which a small organic molecule can activate different substrates and simultaneously control the reaction outcome. In fact, similar types of imidazolidinones have been employed as catalysts for multiple Diels–Alder-type cycloadditions. A direct application of this organocatalytic cycloaddition was described soon after the first report of MacMillan, demonstrating that iminium-ion activation could also be employed to develop intramolecular Diels–Alder reactions (IMDA) [81]. This type of cycloaddition represents a remarkable application of the Diels–Alder reaction that can easily provide a high degree of chemical complexity with high atom-economy and excellent stereocontrol [82]. The unsaturated aldehyde **57** underwent cyclization in the presence of the imidazolidinone catalyst **58** to provide functionalized cyclic compound **59** with a high degree of stereoselection. Interestingly, this new transformation furnished the necessary intermediate for the asymmetric 19-step synthesis of the marine metabolite Solanapyrone D, a phytotoxic polyketide isolated from a fungus (Figure 11) [83].

**Figure 12:**
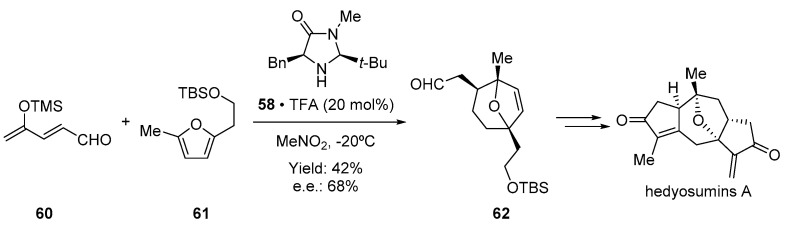
Application of (3 + 2) cycloaddition in the synthesis of hedyosumins A.

The use of this type of catalyst is not limited to the Diels–Alder reaction. In fact, other cycloadditions have been successfully activated and controlled by similar imidazolidinones. Therefore, the electronic equivalent 1,3-dipolar cycloaddition was described soon after by MacMillan and coworkers [84]. Importantly, conveniently designed aldehydes bearing a masked enolate can be used as the dipole in a (4 + 3) cycloaddition [85]. In fact, this methodology has been used during the synthesis of hedyosumins A, B, and C, natural products belonging to the family of guaianolides (Figure 12). The oxabicyclic molecule **62** containing three stereogenic centers is synthesized in a single step by taking advantage of the aforementioned organocatalytic cycloaddition developed by Hamada and coworkers [86]. To this end, dienal **60** and furane **61** were reacted in the presence of Macmillan catalyst **58** to obtain the cycloadduct **62**, which was subjected to further manipulation to deliver the desired natural product.

**Figure 13:**
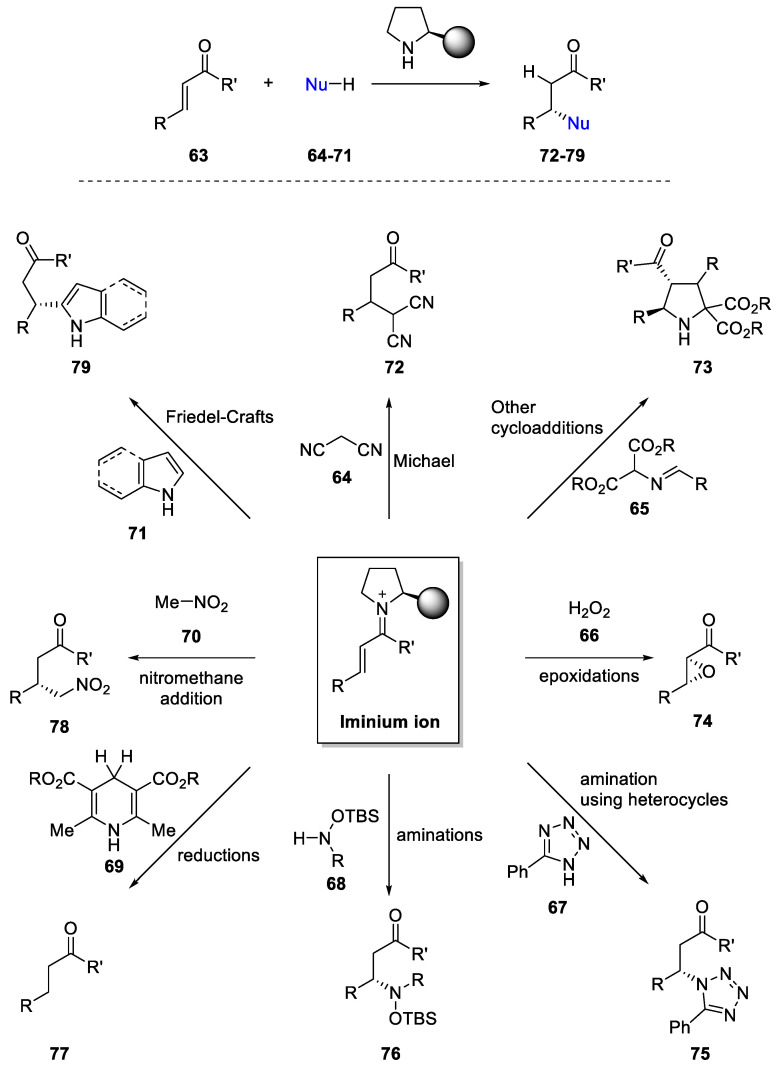
Organocatalytic transformations involving iminium activation of α,β-unsaturated ketones and aldehydes.

Together with Diels–Alder reactions and the other cycloadditions mentioned before, the iminium-activation protocol has been extensively developed over the last two decades [87]. In fact, the iminium ion generated in these processes can be used as a simple electrophile at β-position instead of a dienophile for reacting with different nucleophiles (**64–71**). Therefore, over α,β-unsaturated ketones and aldehyde **63** addition of activated methylene compounds, azomethine ylides, epoxidation, aza-Michael additions, reductions, Michael additions, Friedel–Crafts, and other conjugate additions have been developed with success (Figure 13).

**Figure 14:**
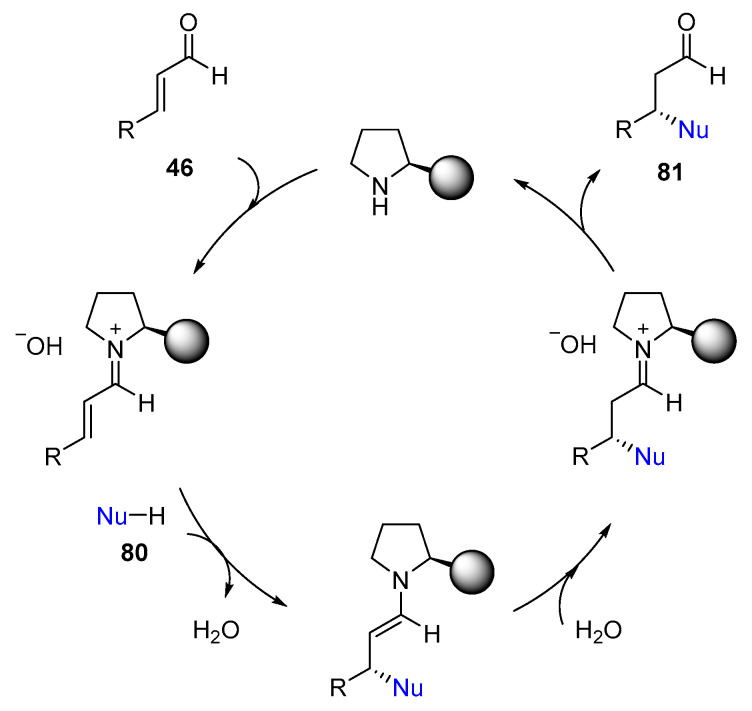
Catalytic cycle associated with iminium-ion activation.

For all of these previous examples, a similar catalytic cycle is proposed: Initially, the reversible covalent condensation between a chiral aminocatalyst (primary or secondary amine) and the α,β-unsaturated aldehyde **46** (also a ketone) leads to the formation of an iminium-ion intermediate, where the LUMO energy of the π system is lowered compared to that of the starting aldehyde. This led to an enhanced reactivity for nucleophilic attack of **80** at the β position of the α,β-unsaturated aldehyde (Figure 14).

**Figure 15:**
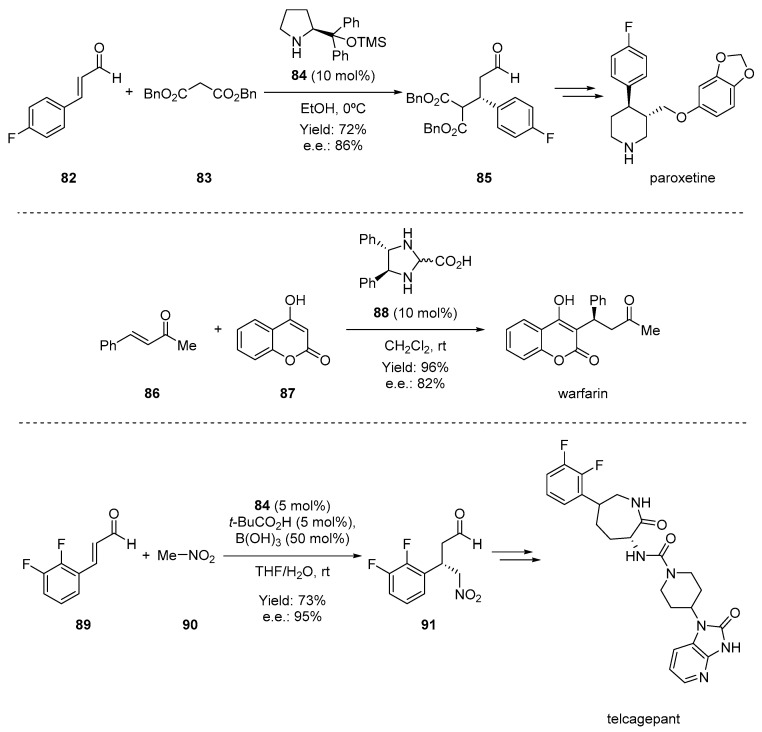
Organocatalytic Michael reactions allowing the synthesis of paroxetine, warfarin, and telcagepant.

The employment of iminium-ion activation in the synthesis of a variety of natural products and pharmaceuticals makes this methodology an interesting tool in asymmetric synthesis (Figure 15). For example, Jørgensen and coworkers employed iminium-ion activation to achieve the enantioselective synthesis of the serotonin reuptake inhibitor (-)-paroxetine [88]. To build the phenyl piperidine core with the two *trans*-disposed substituents at C3 and C4, the authors developed an organocatalytic conjugate addition of malonate **83** to the α,β-unsaturated aldehyde **82** catalyzed by catalyst **84** (Figure 15. In particular, the conjugate addition product **85**, obtained in high yield and e.e., allowed the preparation of (-)-paroxetine in only 6 steps, compared to the 12–14 steps required for the earlier, more conventional syntheses. Similarly, an organocatalyzed strategy allowing the highly economical synthesis of optically active warfarin was developed by Jørgensen and coworkers [89]. Warfarin is one of the most used anticoagulants but is prescribed as a racemate despite that the (*S*)-enantiomer is more active than the (*R*)-enantiomer, while the two enantiomers also have different pharmacokinetic profiles [90]. For the synthesis of optically active warfarin, the authors developed a straightforward Michael addition of 4-hydroxycoumarin **87** to benzylideneacetone **86** catalyzed by **88** that led to the target compound with high yield and e.e. Interestingly, the reaction also proceeds on a kilogram scale and with a possible recycling of the catalyst. Researchers at Merk reported the first application of iminium organocatalysis on an industrial scale in the synthesis of telcagepant [91]. This molecule is a calcitonin gene-related peptide receptor antagonist developed for the treatment of migraine [92]. For the synthesis of this drug, they developed an asymmetric 1,4-addition of nitromethane **70** to α,β-unsaturated aldehyde **89** using the Jørgensen–Hayashi-type catalyst **84**. The key to the success of this organocatalytic transformation was the identification of a dual-acid cocatalyst system, *t*-BuCO_2_H/B(OH)_3_, essential to achieve a high reaction rate and conversion.

## 5. PTC Catalysis

**Figure 16:**
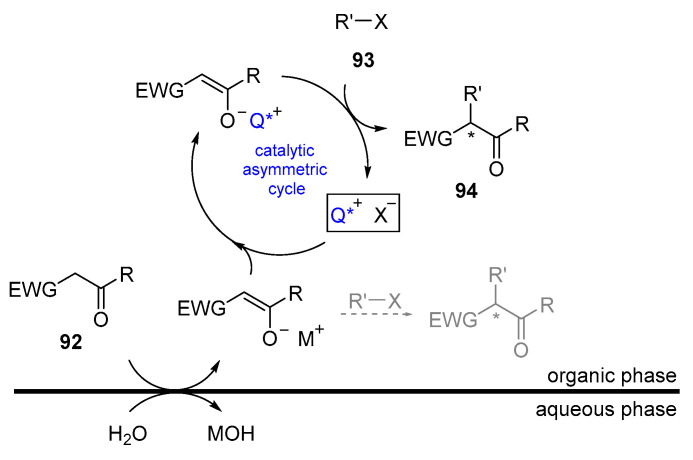
Catalytic cycle for PTC alkylation.

The use of small organic molecules to catalyze organic transformations in a biphasic system is known as “phase transfer catalysis” or PTC [55,56,57,58,92]. It is well-accepted that a small quantity of substance, the catalyst, can transfer one of the substrates from one phase to another, promoting its deprotonation and thereby favoring the reaction. Although the exact mechanism is not completely clear, a plausible mechanism is depicted in Figure 16 [93,94]. The catalytic cycle for the simple alkylation of an activated methylene compound **92** begins with the deprotonation in the aqueous phase by an inorganic salt (the phase transfer catalyst participates in transporting the substrate to the interphase). Then, a metal–catalyst interchange occurs, followed by the alkylation of the generated enolate. This step occurs faster than the direct alkylation of the metal-based enolate. In case that the catalyst is chiral, asymmetric induction can be observed during the reaction outcome.

**Figure 17:**
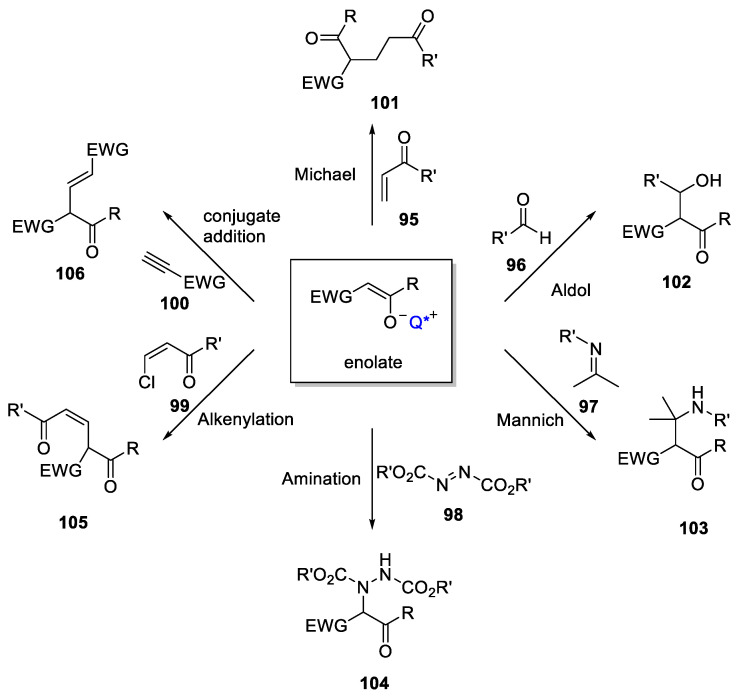
PTC involving alkylation of chiral enolates.

Following a similar mechanism, different reactions have been presented during the last decades. In fact, the most significant advances in this type of catalysis are due to the use of the different electrophiles (**95**–**100**) in the original reaction as well as the use of various active methylene compounds. Thus, Michael acceptors, aldehydes, imines, azoderivatives, or haloalkenes have been used with success for alkylating the corresponding enolates (Figure 17).

**Figure 18:**
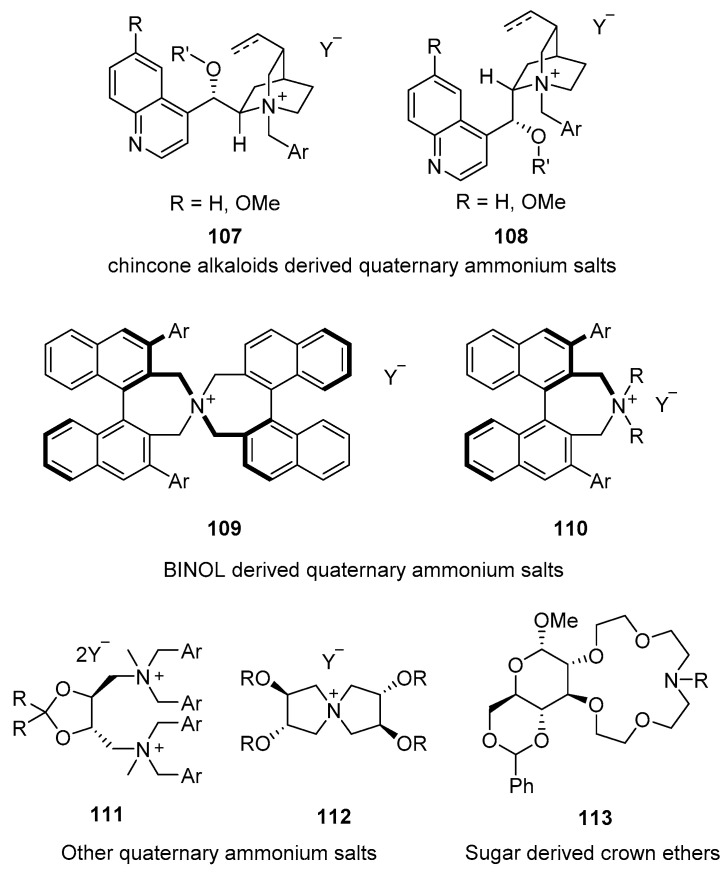
Selected catalysts for PTC.

The catalysts used for performing this type of reaction are, in general, based on quaternary ammonium salts (Figure 18). The cinchone-derived alkaloids **107**–**108** can easily be modified to generate a chiral environment for the reaction process. Alternatively, BINOL-derived ammonium salts **109**–**110** have been extensively used for many transformations. Other quaternary ammonium salts derived from naturally occurring chiral sources such as **111**–**112** have also been studied in limited reactions. Finally, crown ethers **113** derived from different sugars have been employed with success in several transformations.

**Figure 19:**
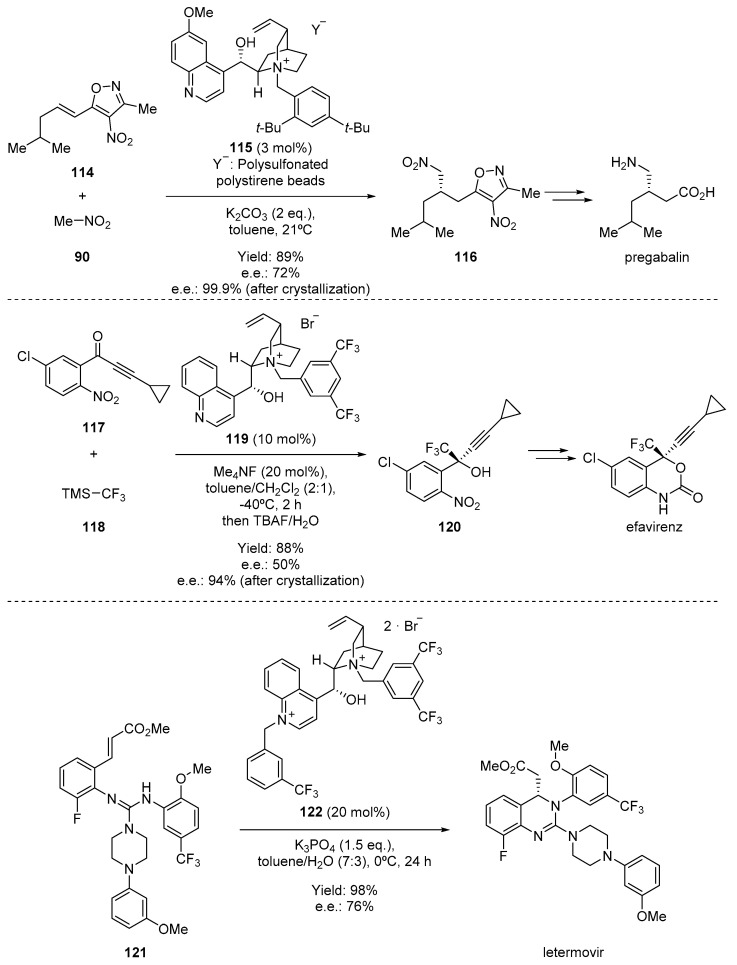
PTC-based transformation, allowing the synthesis of pregabalin, efavirenz, and letermovir.

Using PTC, several pharmaceuticals have been successfully synthesized (Figure 19). For example, nitronate derived from nitromethane-anion can be successfully alkylated with the masked Michael acceptor **114** in the presence of a cinchonidine-derived quaternary ammonium salt **115** as the key step during the asymmetric synthesis of pregabalin, an anticonvulsant and anxiolytic drug originally manufactured by Pfizer [95]. However, PTC is not limited to the alkylation of enolates derived from activated methylene compounds. In fact, this type of catalysis has been extended towards the activation of other nucleophiles, as demonstrated in the synthesis of efavirenz [96]. In this case, a quinine-derived quaternary ammonium salt **119** is used to catalyze the addition of trifluoromethyltrimethylsilane **118** to the enone **117**, which was obtained with good yield and stereoselectivity and could be transformed into the final antiretroviral drug in several steps. Similarly, intramolecular aza-Michael-type reactions can be easily performed on a substrate such as **121** during the preparation of letermovir, an antiviral that acts by inhibiting the CMV DNA terminase complex which is required for viral replication and survival [97].

## 6. H-Bond Catalysis

**Figure 20:**
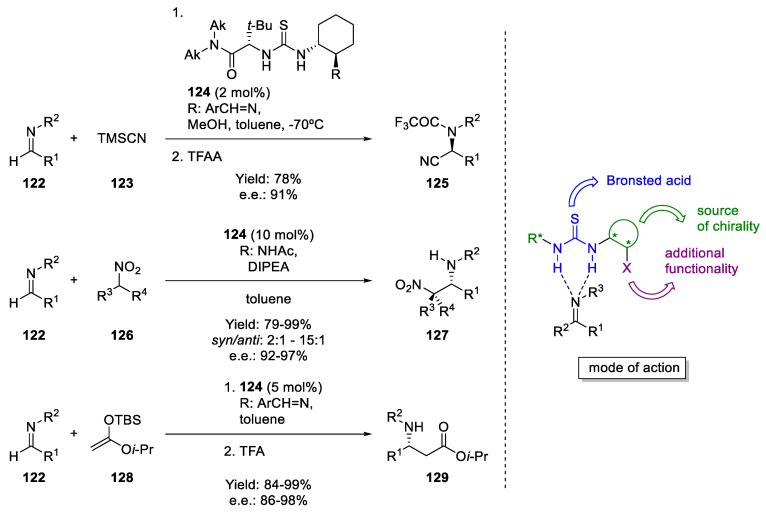
Mode of action of thiourea catalysts (**right**) and their catalytic activity in 1,2-additions to aldimines (**left**).

In contrast to covalent organocatalysis, noncovalent organocatalysis has mainly been developed as H-bond and Brønsted acid catalysis. H-bonds, despite their function as a structural determinant, play a crucial role by inducing the acceleration of a myriad of biochemical reactions. A wide range of enzymes employ this principle to activate a given substrate, including serine proteases, type II aldolases, and others [98]. Electrophilic activation by chiral small-molecule H-bond donors was discovered in pioneering studies beginning in the mid 1980s, but it emerged as an important paradigm for enantioselective catalysis only after the seminal reports by Jacobsen [11] and Corey [99] that independently reported an asymmetric Strecker reaction using H-bonding organocatalysts. Mechanistic studies of Jacobsen’s reaction revealed that the catalytic activity can be attributed to the ability of the thiourea to efficiently activate the electrophile (i.e., the imine) for nucleophilic attack by engaging the imine via double H-bonding, while the high enantioselectivity arises from the significant steric effect of the amide portion of the catalyst (Figure 20, right) [100]. Over the last 20 years, the ability of H-bond donors to catalyze useful organic transformations has allowed the identification of a remarkable number of new enantioselective reactions together with the design of novel catalyst frameworks [13,32,54,57,98,101]. Thioureas and diols have been the most extensively applied H-bonding catalysts. In particular, thiourea-based catalysts have shown the ability to promote highly enantioselective cyanosilation [11], Henry [102], Mukaiyama–Mannich [103], and other reactions, with excellent reaction performance (Figure 20, left).

**Figure 21:**
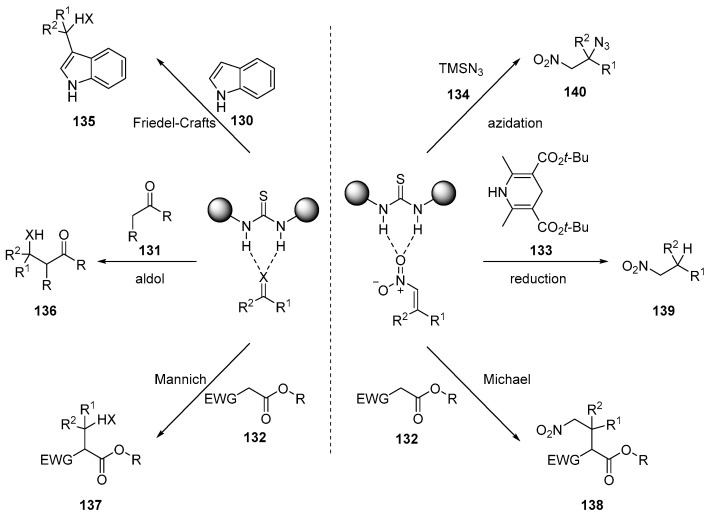
Thiourea-catalyzed addition to carbonyl compounds and nitroalkenes.

Not only aldimines but also other carbonyl-containing compounds have been used as acceptors or as donors in several reactions, including Friedel–Crafts, aldol, and Mannich reactions. Additionally, nitroalkenes and other electron-deficient alkenes have been utilized as Michael acceptors in conjugate additions in Michael reactions (Figure 21). 

**Figure 22:**
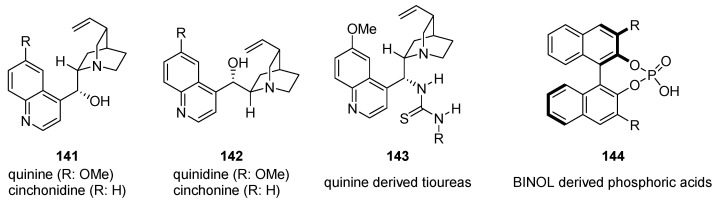
Selected catalysts for hydrogen-bonding activation.

**Figure 23:**
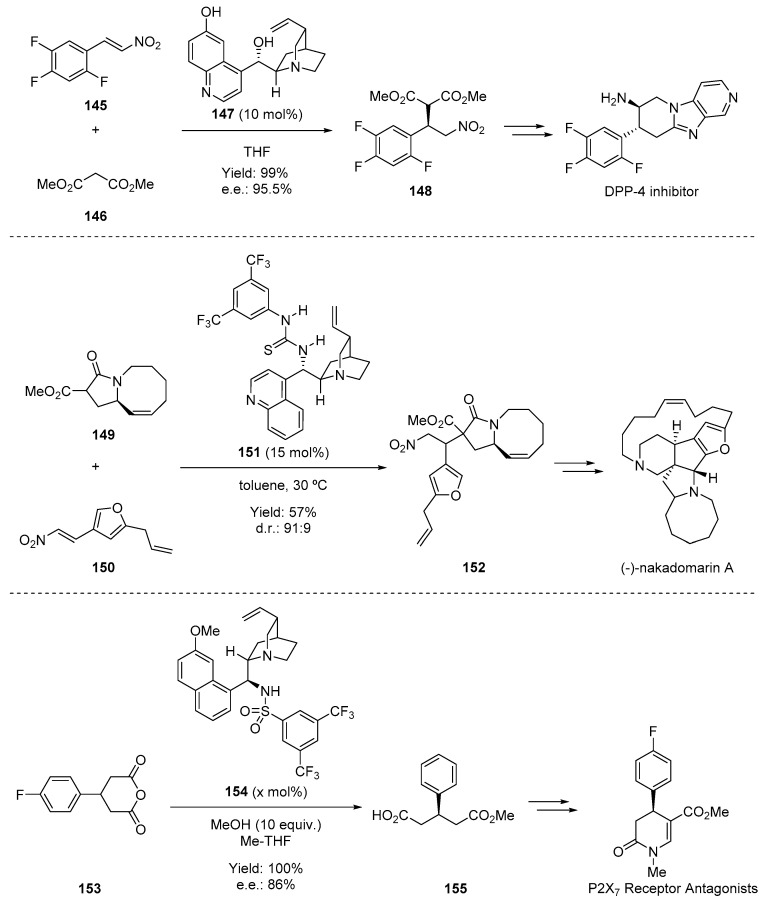
H-bonding catalysts used for the synthesis of the DPP-4 inhibitor, nakadomarin A, and the P2 × _7_ receptor antagonist.

An exceptionally useful class of bifunctional organocatalysts is represented by the cinchona-derived thiourea organocatalyst family (Figure 22) [101]. Quinine, quinidine, cinchonine, and cinchonidine (**141**–**142**) are natural products extracted from the bark of *Cinchona officinalis*. From a structural point of view, these analogues are highly complex, presenting a basic and nucleophilic quinuclidine and a quinoline unit joined together by two C-C single bonds, a secondary alcohol, and five stereocenters (Figure 22). These alkaloids can easily be modified [104,105]. Among the modified structures, cinchona-thiourea organocatalysts have assumed remarkable importance. In these catalysts, the quinuclidine tertiary nitrogen is basic, while the thiourea at C-9 activates the approaching electrophile through H-bonding interactions and the relative orientation of the quinoline and quinuclidine rings creates the chiral pocket responsible for the stereochemical outcome. Catalyst **143** is one of the most widely used cinchona-thiourea catalysts, that offers several beneficial properties [106,107]. This catalyst has been successfully employed to promote a wide range of synthetically useful transformations, including 1,4-additions, Mannich, Henry, and cycloaddition reactions, some of which are depicted in Figure 23 [93]. In the field of Brønsted acid catalysis [108,109], a variety of different catalyst classes have emerged in recent years, one of the most prominent being BINOL-derived phosphoric acids (Figure 22) [110,111]. These catalysts are very effective at lowering the LUMO of an electrophile, thereby activating the substrate towards nucleophilic attack. BINOL-derived catalysts have achieved such success thanks to their unique characteristics: (a) conformational rigidity thanks to the formation of the seven-member ring that fixes the conformation of the acid, (b) a Lewis basic site located next to the acidic proton, and (c) 3,3′-substituents that influence the yields and the stereoselectivity of these reactions [112]. Since the seminal papers of Akiyama [113] and Terada [114], these catalysts have been successfully applied in a variety of enantioselective reactions [115,116]. 

H-bonding catalysis has been employed in the synthesis of natural products and biologically active compounds. H-bonding catalysis has been successfully employed to synthesize diverse natural products and pharmaceuticals. Researchers at Merck employed a cinchona catalyst to synthesize the aminopiperidine-fused imidazopyridine dipeptidyl peptidase IV (DPP-4) inhibitor (Figure 23. They developed an enantioselective Michael addition between dimethylmalonate **146** and nitrostyrene **145** catalyzed by demethylquinidine **147** [115]. The desired product (**148**) was obtained in 95.5% e.e., with the enantioselectivity of the reaction strongly dependent on both the reaction temperature and the water content. A remarkable application of the cinchona-thiourea catalyst is shown during the key step of the total synthesis of (-)-nakadomarin A, a marine alkaloid of the manzamine family [116]. In this case, bifunctional catalyst **151** induces the diastereoselective Michael addition of **149** to **150** in good yield as a 91:9 mixture of diastereomers. The final example illustrates the application of an asymmetric methanolysis developed at Roche for the synthesis of 3,4-dihydropyridin-2-one derivatives, the core structures of P2X7 receptor antagonists [117]. In this case, catalyst **151** again turned out to induce the formation of **155** from **153** in 80% e.e., which was used in the pilot-plant synthesis. The authors also investigated de-symmetry reactions using the more efficient sulfonamide catalyst **154**, which produced **155** in a higher e.e. (86%) under more convenient reaction conditions.

## 7. Conclusions

Asymmetric organocatalysis has undergone a sensational development in the last two decades since it went from its discovery to global recognition with the awarding of the Nobel Prize. This development has brought this technique to be considered, in all aspects, at the same level as enzymatic and metal catalysis for synthesizing enantiopure molecules. As a result, general interest in asymmetric organocatalysis has moved beyond mere “methodology” development to a much more “applied” field, such as application as a key step in the synthesis of various biologically active or medicinal chemistry-relevant molecules. Indeed, several advantages of this methodology, such as the absence of metal contaminants, operational simplicity, availability of catalysts, and the possibility of a wide range of possible reactions, make it very attractive to medicinal chemists. As discussed in this review, there are many ways of activating organic molecules, and plenty of reactions have been developed in an organocatalytic fashion. In parallel, the applications of organocatalysis, both academic and industrial, in the field of the synthesis of natural products, drugs, and drug candidates have increased year by year. In the present review, we only covered a small part of the available literature and, based on this, we strongly believe that asymmetric organocatalysis will be, over the next years, increasingly used by medicinal chemists to access enantiopure molecules in an easy, cheap, and environmentally friendly manner. This process will be supported and facilitated by the many developments in organocatalysis that will further increase the possible applications of this incredible technique.

## Data Availability

Not applicable.
